# Comparison of Radiological and Postoperative Tumor Volume Measurements for SIOP Risk Stratification in Wilms Tumord

**DOI:** 10.3390/diagnostics16172698

**Published:** 2026-08-24

**Authors:** Aseel Hamdan, Azza Gharaibeh, Rula Al-Qawabah, Khalil Ghandour, Iyad Sultan, Hadeel Halalsheh

**Affiliations:** 1 Department of Pediatrics, King Hussein Cancer Center, Al-Jubaiha P.O. Box 1269, Amman 11941, Jordan; ah.11864@khcc.jo (A.H.); isultan@khcc.jo (I.S.); 2 Department of Diagnostic Radiology, King Hussein Cancer Center, Amman 11941, Jordan; ag.12717@khcc.jo (A.G.); ra.14467@khcc.jo (R.A.-Q.); 3 Department of Surgery, King Hussein Cancer Center, Al-Jubaiha P.O. Box 1269, Amman 11941, Jordan; jafapal48@yahoo.com; 4 Department of Pediatrics, University of Jordan, Amman 11941, Jordan; 5 Artificial Intelligence Office, King Hussein Cancer Center, Amman 11941, Jordan

**Keywords:** Wilms tumor, nephroblastoma, SIOP protocol, tumor volume, risk stratification, ellipsoid formula, planimetry, Bland–Altman analysis

## Abstract

**Background:** Tumor volume in Wilms tumor (WT) guides risk stratification under the International Society of Pediatric Oncology (SIOP) protocol. While the radiological ellipsoid formula is the current protocol-specified reference method, modalities such as planimetry, intraoperative specimen weight, and pathological volume are often used interchangeably in practice without systematic evaluation. **Methods:** We reviewed 60 pediatric WT patients. Three measurements were compared against the SIOP-standard radiological ellipsoid volume: radiological planimetry, intraoperative specimen weight, and pathological tumor volume. Pearson and Spearman correlations and Bland–Altman analysis were performed. **Results:** Radiological planimetry showed near-perfect correlation with the SIOP ellipsoid (Pearson r = 0.993, *p* < 0.001; mean bias +21.1 cm^3^, 95% limits of agreement −82.8 to +124.9 cm^3^, percentage bias +6.1%). Intraoperative specimen weight (r = 0.730) and pathological tumor volume (r = 0.594) showed moderate-to-good correlations but wide limits of agreement and systematic biases of +46.6% and −34.1%, respectively. At the critical 500 cm^3^ treatment threshold, ellipsoid and planimetry showed 100% concordance. However, using pathology volume would have produced discordant (lower) risk categorization for 47% of ellipsoid-defined high-risk patients, a discordance whose clinical treatment implications were not directly assessed in this study. Conversely, relying on intraoperative weight would have upstaged 9.1% of patients to a higher risk category, which could correspond to a more intensive treatment category, including doxorubicin, under the SIOP protocol; whether this would translate into unnecessary treatment in practice was not directly assessed in this study. **Conclusions:** Radiological planimetry is a highly reliable surrogate for the SIOP ellipsoid formula across all stages and histologies. Intraoperative weight and pathological volume exhibit significant systematic biases and should not be used to revise radiological risk groups. These findings support continued anchoring of SIOP risk stratification to preoperative radiological volumetry, consistent with the currently validated protocol threshold, to avoid unintended deviations in treatment intensity.

## 1. Introduction

Wilms tumor (WT) is the most common pediatric renal malignancy, accounting for approximately 6% of all childhood cancers [1,2]. Following five decades of cooperative group research, survival for localized disease now exceeds 90%, a remarkable achievement that has been accomplished alongside progressive reductions in treatment intensity for most patient subgroups [3]. A central contributor to these outcomes has been the development of risk-adapted treatment strategies that accurately identify patients requiring intensification while sparing low-risk patients from unnecessary toxicity.

The International Society of Pediatric Oncology Renal Tumor Study Group (SIOP-RTSG) has since 1971 championed a strategy of preoperative (neoadjuvant) chemotherapy before nephrectomy [4,5,6,7]. This approach reduces tumor volume, lowers the risk of intraoperative rupture, and provides a selection advantage through the response to chemotherapy, which itself carries prognostic information [5]. Under the current SIOP 2001 and UMBRELLA SIOP-RTSG 2016 protocols, postoperative treatment is stratified according to the histological risk group, comprising low, intermediate, and high risk based on the proportion of chemotherapy-induced necrosis and blastemal component, and tumor stage [6,8,9]. Residual tumor volume measured on imaging after neoadjuvant chemotherapy and immediately before surgery is an additional prognostic parameter: a threshold of 500 cm^3^ in the intermediate-risk histological group has been shown to discriminate significantly between outcome groups in the SIOP 93-01 and SIOP 2001 datasets [10,11].

Postchemotherapy presurgical tumor volume is routinely measured from cross-sectional imaging, computed tomography (CT) or magnetic resonance imaging (MRI), obtained after completion of neoadjuvant treatment. This is performed using one of two methods: the ellipsoid formula (V = π/6 × L × W × H, where L, W, and H are the maximum orthogonal diameters) or planimetric volumetry (summation of cross-sectional tumor areas across serial axial slices) [12,13]. The ellipsoid formula is mathematically simpler and more widely implemented in routine clinical practice; planimetry is more labor intensive but potentially more precise for irregularly shaped tumors [14]. Prior studies have compared ellipsoid and segmentation-based volumetry in WT, but the degree to which the ellipsoid formula and planimetry agree specifically for SIOP risk-threshold classification, and whether the choice of method could reclassify a patient across the 500 cm^3^ threshold, have not been directly characterized.

In addition to radiological measurement, intraoperative specimen weight and postoperative pathological volume are routinely recorded in many centers, raising the question of whether these measures might provide additional or alternative stratification information [15,16,17]. Given that the radiological volume is measured after chemotherapy-induced tumor change, its relationship with postoperative pathological measurements of the same residual tumor is expected to reflect both biological and technical differences between in vivo imaging and ex vivo specimen assessment. Understanding this relationship is important to ensure that postoperative measurements are not inadvertently used to revise risk classification in a manner inconsistent with the SIOP framework.

The aim of this study was therefore to compare all available volumetric measurement modalities—postchemotherapy presurgical ellipsoid formula, postchemotherapy presurgical planimetry, intraoperative specimen weight, and postoperative pathology volume—in a cohort of children with WT managed under the SIOP protocol. Our primary objective was to determine which alternative method demonstrates the closest agreement with the ellipsoid standard. Secondary objectives included evaluating Bland–Altman agreement metrics, identifying systematic biases, and assessing whether agreement varies by disease stage or histological subtype.

## 2. Methods

### 2.1. Study Design and Patients

This was a retrospective, single-center study of children diagnosed with unilateral WT and treated at King Hussein Cancer Center (KHCC) in Jordan. Patients diagnosed between 2020 and 2024 who had available preoperative cross-sectional imaging and surgical/pathological specimen data were considered eligible. The study was approved by the KHCC Institutional Review Board (25KHCC006).

Data were extracted from both electronic and paper medical records by clinical staff who were blinded to the radiological volume calculations to prevent bias. Extracted data included patients’ demographics (age, sex), tumor characteristics (histologic subtype, staging), intraoperative tumor weight, pathology tumor weight, and pathology tumor volume. Two pediatric radiologists (A.G. and R.Q.) reviewed by consensus all preoperative CT scans. They calculated both the ellipsoid tumor volume and the planimetry tumor volume.

### 2.2. Tumor Volume Measurement

Four distinct measurements were documented for comparison in the primary analysis:

Radiological ellipsoid volume: Maximum tumor dimensions in three orthogonal planes were measured on axial CT imaging obtained after completion of neoadjuvant chemotherapy and immediately before surgery. Volume was calculated as V = (π/6) × L × W × H (cm^3^), where L, W, and H represent length, width, and craniocaudal height [14].

Radiological planimetric volume: Planimetry was performed on the same postchemotherapy presurgical imaging study. Tumor area was traced manually on each axial slice, and the areas were summed using an external application (syngo.via.) to estimate total volume (cm^3^). All radiological measurements (ellipsoid and planimetry) in this cohort were obtained from axial CT imaging; MRI was not used for volumetric assessment in this series. Acquisition and reconstruction parameters, including slice thickness and reconstruction interval, followed standard departmental CT protocols but were not individually recorded for each examination in the retrospective medical records and could therefore not be reported per patient (see the limitations noted in Section 4).

Intraoperative specimen weight: The resected nephrectomy specimen was weighed immediately after removal in the operating theatre, recorded in grams (g) by the operating surgeon; these values were extracted from surgical records.

Postoperative pathology volume: The tumor specimen was measured by the reporting pathologist in three dimensions, and volume was calculated using the ellipsoid formula applied to the pathological specimen (cm^3^) and was extracted from the pathology reports; because these data were abstracted from retrospective records, whether measurement occurred before or after formalin fixation was not consistently documented (see the limitations noted in Section 4).

As a secondary exploratory objective, we analyzed pathological specimen weight (the resected specimen weighed by the pathologist before gross sectioning) where available. Due to inconsistent documentation (50% missingness), this metric was excluded from the primary agreement analysis and reserved for exploratory correlation review to evaluate its potential as a proxy for volume.

Throughout this manuscript, “intraoperative specimen weight” refers to the fresh nephrectomy specimen weighed in the operating theatre, “pathological specimen weight” refers to the specimen weighed by the pathologist before gross sectioning, and “pathological tumor volume” refers to the ellipsoid formula volume calculated from the pathologist’s three-dimensional measurements of the tumor specimen.

### 2.3. SIOP Risk Stratification

In accordance with the SIOP protocol, radiological tumor volume measured by the ellipsoid formula on postchemotherapy presurgical imaging was the measurement used for clinical decision-making. A volume exceeding 500 cm^3^ was designated high-risk for adjuvant treatment planning purposes, as validated in the SIOP 93-01 and SIOP 2001 trial datasets [10,11]. The direct clinical application of this threshold within the UMBRELLA SIOP-RTSG 2016 protocol is confined to the intermediate-risk, non-stromal, non-epithelial histological subgroup; in the present analysis, the 500 cm^3^ cut-point was applied across the full cohort as a measurement-comparison benchmark rather than as a simulation of protocol-driven treatment allocation for every patient.

### 2.4. Statistical Analysis

Agreement with the SIOP ellipsoid was assessed using multiple statistical approaches. Initial correlation analysis included Pearson correlation coefficients (r) with 95% confidence intervals (CI), complemented by Spearman rank correlation (rho) to account for non-parametric distributions. Distributional assumptions for Pearson correlation were assessed by visual inspection of scatterplots; Spearman rank correlation was additionally reported given evidence of non-normality and influential outliers in some comparisons.

Bland–Altman analysis was also utilized, where differences between each method and the SIOP ellipsoid were plotted against their mean. For this assessment, the mean bias, standard deviation (SD) of differences, and 95% limits of agreement (LoA = mean ± 1.96 × SD) were calculated [18]. Percentage bias was defined as the mean of individual percentage differences relative to the pairwise mean.

Subgroup analyses were conducted to determine Pearson correlations between the ellipsoid and planimetry methods within specific strata of disease stage (I–IV) and histological subtype. Risk reclassification relative to the 500 cm^3^ threshold was assessed by cross-tabulating categorical agreement for each alternative method against the ellipsoid reference and is summarized descriptively in Section 3. All statistical analyses were performed in R version 4.4.3 (R Foundation for Statistical Computing).

## 3. Results

### 3.1. Cohort Characteristics

Sixty patients with histologically confirmed WT were included. Median age at diagnosis was 3.7 years (range 0.6–15.2). The cohort included patients across SIOP stages I-IV and varied histological subtypes, primarily consisting of favorable histology (FH; *n* = 42), followed by blastemal type (BT; *n* = 10), diffuse anaplasia (DA; *n* = 5), and focal anaplasia (FA; *n* = 3). Detailed characteristics are presented in Table 1.

### 3.2. Volume Data Availability

Postchemotherapy presurgical tumor volume was available by both ellipsoid formula and planimetry in all 60 patients. Postoperative pathology volume was available in 54 patients (90%), and intraoperative specimen weight in 55 patients (92%). All radiological measurements were performed on imaging obtained after completion of neoadjuvant chemotherapy and immediately before nephrectomy. Pathological specimen weight was documented for only 30 patients (50%) across the study period; consequently, this specific measure was excluded from the primary analysis and reserved for exploratory review. Descriptive statistics are presented in Table 2.

### 3.3. Postchemotherapy Presurgical Tumor Volumes and SIOP Risk Stratification

Median postchemotherapy presurgical tumor volume was 201 cm^3^ (range 16–1736) by ellipsoid formula and 232 cm^3^ (range 13–1952) by planimetry (Table 2). Fifteen patients (25%) were classified as high-risk (>500 cm^3^) by ellipsoid formula and 15 (25%) by planimetry.

### 3.4. Agreement Between Postchemotherapy Radiological Measurement Methods

**Figure 1:**
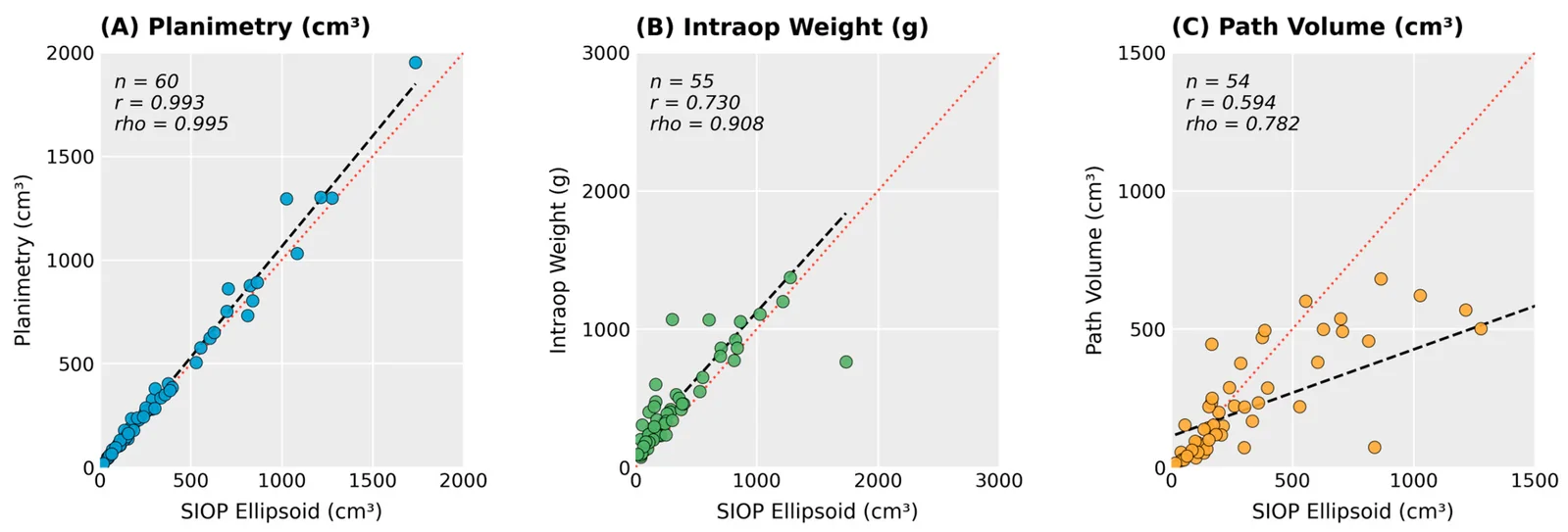
Scatter plots of the SIOP ellipsoid volume (*x*-axis, cm^3^) versus each alternative measurement method (*y*-axis). (**A**) Radiology planimetry (cm^3^), (**B**) intraoperative specimen weight (g), (**C**) pathological tumor volume (cm^3^). Dashed black lines: linear regression fits. Dotted red lines: line of identity.

**Figure 2:**
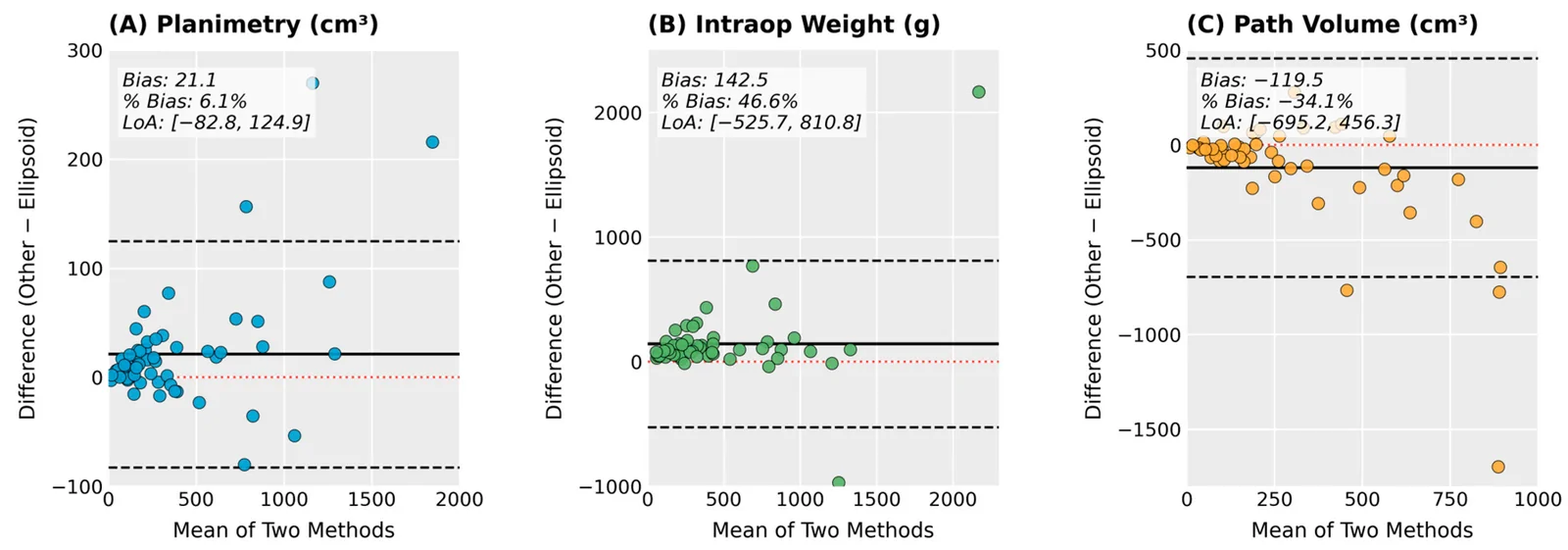
Bland–Altman plots comparing each method with the SIOP ellipsoid. The *y*-axis shows the difference calculated as alternative method minus ellipsoid volume. Solid horizontal line: mean bias. Dashed lines: 95% limits of agreement. Dotted red line: zero. (**A**) Planimetry (cm^3^), (**B**) intraoperative specimen weight (g)—shown against the ellipsoid volume (cm^3^) as an exploratory, numerical (not unit-matched) comparison, since specimen weight and radiological volume are not the same physical quantity, (**C**) pathological volume (cm^3^).

Ellipsoid formula and planimetry demonstrated near-perfect correlation for postchemotherapy presurgical tumor volume (r = 0.993, 95% CI: 0.988–0.996; Spearman rho = 0.995), indicating a strong linear association between the two methods (Table 3; Figure 1). Bland–Altman analysis revealed that planimetry had the smallest mean bias (+21.1 cm^3^, SD = 53.0) and the narrowest 95% LoA (−82.8 to +124.9 cm^3^), with a percentage bias of only +6.1% (Figure 2). This means planimetry slightly overestimates the ellipsoid, consistent with planimetry capturing irregular margins that the three-diameter approximation misses.

For SIOP risk stratification, none of the 60 patients were classified differently between the two methods, yielding a risk-category concordance of 100% (exact Clopper–Pearson 95% CI for the discordance proportion: 0.0–6.0%). The two methods produced identical threshold classifications in this cohort; however, with only 15 patients above the 500 cm^3^ threshold, this finding does not exclude clinically meaningful discordances in tumors close to the cut-point. This pattern is best characterized as close agreement rather than measurement equivalence, and validation in a larger cohort enriched for borderline volumes is warranted before broader claims of interchangeability can be made.

### 3.5. Radiological Versus Postoperative Pathology Volume

The ellipsoid radiological method yielded substantially higher volume estimates than the postoperative pathology volume of the same tumor. This discrepancy reflects differences between in vivo radiological measurement of the tumor in situ and ex vivo pathological measurement of the resected specimen. Ellipsoid versus pathology (n = 54 pairs) had the weakest overall association (r = 0.594, 95% CI: 0.388–0.743; rho = 0.782, Table 3, Figure 1). Bland–Altman analysis revealed that pathological tumor volume underestimated the ellipsoid volume by 119.5 cm^3^ on average (SD = 293.8), with LoA of −695.2 to +456.3 and a percentage bias of −34.1% (Figure 2). This negative bias may reflect tissue shrinkage from formalin fixation if measurement occurred after fixation, the difficulty of measuring tumor dimensions on a bisected gross specimen, and ill-defined margins in tumors exhibiting extensive postchemotherapy necrosis; because the timing of pathological measurement relative to fixation was not consistently documented in the retrospective records, the relative contribution of fixation-related shrinkage cannot be determined.

One patient showed a particularly large discrepancy: radiological volume 1736 cm^3^ versus pathology volume 38 cm^3^ (>97% difference), consistent with near-complete chemotherapy response with predominantly necrotic or cystic residual tissue that may have decompressed upon resection.

At the 500 cm^3^ threshold, 7 of the 15 patients (47%) classified high-risk by radiological ellipsoid had postoperative pathology volumes ≤500 cm^3^. Six patients (40%) were classified high-risk by both methods. The remaining two high-risk patients were excluded from this specific comparison due to missing postoperative pathology volume data (n = 54 total). No patient classified as low-risk by radiological measurement had a pathology volume >500 cm^3^. These discordances reflect the systematic difference between radiological and pathological measurement approaches rather than further tumor change after imaging.

### 3.6. Intraoperative Specimen Weight Versus Ellipsoid Volume

Intraoperative specimen weight demonstrated a moderate linear correlation (r = 0.730, 95% CI: 0.576–0.834) but a stronger rank-based correlation (rho = 0.908), suggesting that a few extreme outlier observations attenuate the Pearson coefficient (Table 3, Figure 1). Bland–Altman analysis revealed that specimen weight systematically overestimated the ellipsoid volume by a mean of 142.5 g (SD = 340.9), with very wide LoA (−525.7 to +810.8) and a substantial percentage bias of +46.6%. This is primarily due to the inclusion of non-tumoral tissues (normal parenchyma, fat) in the surgical resection (Figure 2).

One patient had an intraoperative specimen weight of 3250 g with a radiological volume of 1085 cm^3^ (ratio 3.0), likely reflecting abundant cystic or fluid-filled components that contributed to specimen mass but appeared partially decompressed on imaging.

### 3.7. Clinical Impact on Risk Stratification

To assess the clinical impact of these systematic biases, we evaluated how alternative methods would alter protocol-driven risk stratification. Using the UMBRELLA protocol threshold of 500 cm^3^, relying on intraoperative weight rather than the radiological ellipsoid would have resulted in the misclassification (upstaging) of 5 patients (or 9.1% of the 55 patients with available intraoperative specimen weight). Of these five patients, three had true ellipsoid volumes between 300–360 cm^3^. For these individuals, this would have placed them in a higher SIOP risk category, which under the treatment protocol corresponds to more intensive adjuvant chemotherapy, including doxorubicin; whether this would translate into unnecessary cardiotoxic exposure in practice was not directly assessed in this study, as actual treatment decisions and outcomes were not evaluated.

### 3.8. Subgroup Analyses

**Figure 3:**
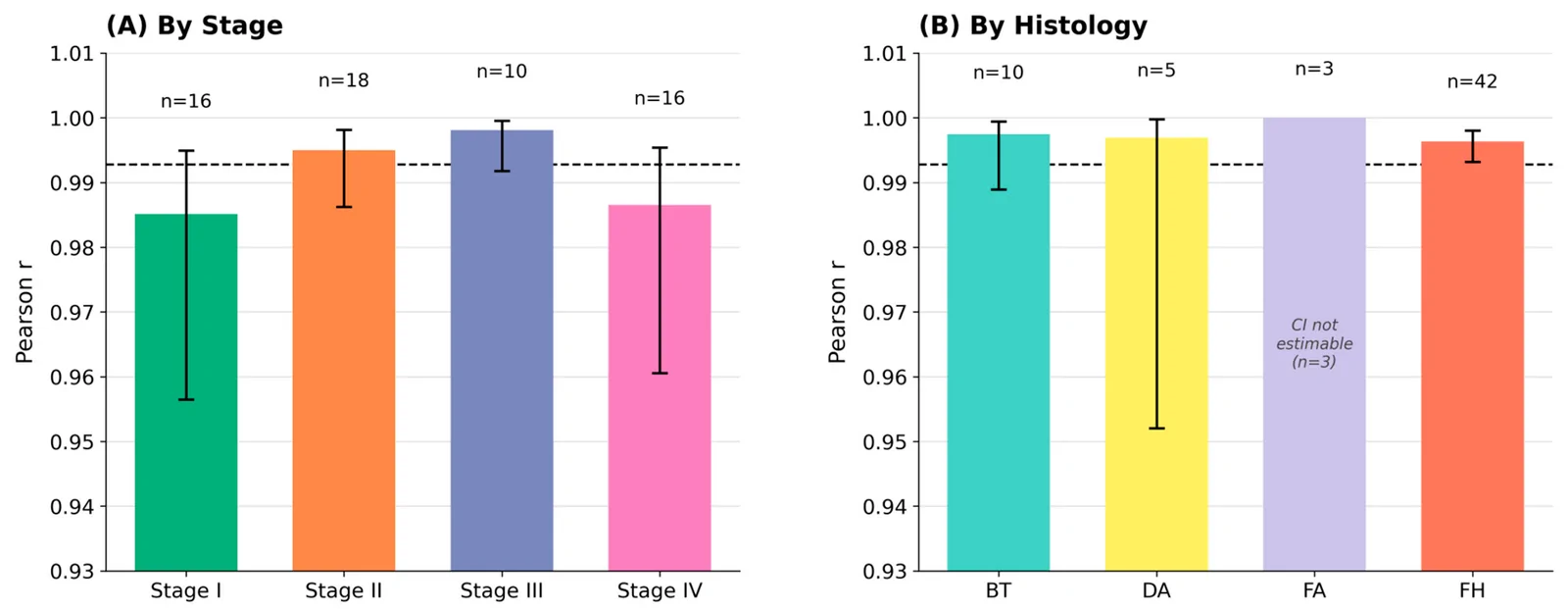
Subgroup correlations between SIOP ellipsoid and radiological planimetry, with 95% confidence intervals (Fisher z-transformation) shown as error bars. (**A**) By SIOP summary stage and (**B**) by histological subtype. Dashed line: overall r = 0.993. A 95% CI could not be estimated for the focal anaplasia subgroup (*n* = 3); the wide or non-estimable intervals for the smallest subgroups (focal anaplasia, *n* = 3; diffuse anaplasia, *n* = 5) indicate that these exploratory estimates are considerably less precise than the near-identical point estimates suggest.

The correlation between ellipsoid and planimetry methods remained uniformly excellent across all stages (r = 0.985–0.998) and histological subtypes (r = 0.996–1.000). Stage III tumors had the highest correlation (r = 0.998, n = 10). The FA subgroup had r = 1.000, though n = 3 limits interpretation (Figure 3, Table 4). These subgroup correlations should be interpreted as exploratory and underpowered given the small sample sizes within several strata (e.g., n = 3 for focal anaplasia, n = 5 for diffuse anaplasia); precision estimates were not calculated for individual subgroups because of these sample-size constraints.

### 3.9. Exploratory: Pathological Specimen Weight

Pathological specimen weight was available for only 30 patients (50%). In this subset, the Pearson correlation with the SIOP ellipsoid was strong (r = 0.951, 95% CI: 0.899 to 0.977, Spearman rho = 0.915). However, the mean bias was +110.7 g (SD = 106.2), with a percentage bias of +48.4%. This indicates that while weight increases proportionally with volume, the absolute values are not interchangeable. Relying on weight measurements would significantly deviate from the established 500 cm^3^ volumetric threshold used for risk stratification.

## 4. Discussion

Accurate tumor volume measurement is central to risk stratification and surgical planning in pediatric Wilms tumor [6,7,9,19]. This study systematically compared all four volumetric measurement modalities available in WT management—the postchemotherapy presurgical ellipsoid formula, postchemotherapy presurgical planimetry, intraoperative specimen weight, and postoperative pathology volume—in a cohort of 60 patients managed under the SIOP protocol. The principal findings are threefold. First, the ellipsoid formula and planimetry produced highly correlated volumes and identical threshold classifications in this cohort, representing close agreement rather than measurement equivalence and supporting the ellipsoid formula’s continued use for SIOP risk stratification; given the modest number of patients near the 500 cm^3^ threshold, confirmation in a larger cohort is warranted before broader claims of interchangeability can be made. Second, postchemotherapy presurgical radiological volume is systematically higher than postoperative pathology volume of the same tumor, reflecting inherent differences between in vivo radiological and ex vivo pathological measurement; this discrepancy does not undermine the validity of radiological volumetry as the SIOP-specified reference method. Third, intraoperative specimen weight, while correlating with radiological volume, cannot serve as a quantitative substitute for radiological volumetry.

The near-perfect agreement between the ellipsoid formula and planimetry with a marginal mean bias demonstrated in this series is consistent with the established accuracy of the ellipsoid formula for approximately spherical tumors in clinical practice [14], and suggests that the geometric regularity of WT post neoadjuvant therapy makes it an ideal candidate for the simplified ellipsoid approximation.

These findings have direct practical implications. Planimetric volumetry is more labor intensive, requires dedicated software, and carries greater potential for inter-observer variability through the subjective process of slice-by-slice delineation [17]. Given the close agreement demonstrated here, the additional resources required for planimetry may not be justified in routine clinical practice. The ellipsoid formula, applied to three orthogonal diameter measurements readily obtainable from any cross-sectional imaging study, is sufficient for SIOP risk stratification. This conclusion supports the approach taken in many national programs where the ellipsoid formula is the standard of care.

The close agreement in volume estimates between ellipsoid and planimetry methods is consistent with previous literature reporting a marginal underestimation by the ellipsoid compared to segmentation-derived volumes. This aligns with Buser et al., who reported an approximate 10% underestimation by the ellipsoid compared to segmentation-derived volumes in 45 WT patients [12]. Our observed 6.1% positive bias for planimetry is of a similar magnitude and logically reflects planimetry’s superior ability to capture geometrically irregular tumor margins that the three-diameter approximation misses. While Muller et al. found a larger 22% underestimation by the ellipsoid relative to expert segmentations, their small sample (17 patients) may have disproportionately featured geometrically complex tumors [13].

Whether this 6% measurement translates into clinical impact depends heavily on the tumor’s proximity to treatment thresholds. For example, for a tumor measuring 480 cm^3^ by the ellipsoid method, a 6% planimetry overestimate yields a volume of approximately 509 cm^3^. This discrepancy could theoretically cross the 500 cm^3^ threshold under the UMBRELLA protocol potentially triggering treatment intensification.

The systematic excess of postchemotherapy radiological volume over postoperative pathology volume (mean bias +119.5 cm^3^) is consistent with well-recognized differences between in vivo and ex vivo tumor measurement. Radiological measurement captures the tumor in its native anatomical context, including surrounding edema, reactive tissue, and residual cystic components that may not be recovered intact at pathological assessment. Pathological measurement of the resected specimen reflects the ex vivo dimensions after surgical removal, tissue handling, and fixation artifact. The patient with a 97% volumetric discrepancy between radiological and pathological estimates most likely represents near-complete chemotherapy response with predominantly necrotic or cystic residual tissue, which may have decompressed or collapsed upon resection. This measurement discrepancy should not be interpreted as a limitation of radiological volumetry. The SIOP protocol uses postchemotherapy presurgical radiological volume for risk stratification because it reflects the residual tumor burden in vivo at the point of surgical decision-making, which is the biologically and clinically relevant quantity for determining adjuvant treatment intensity [5]. The 500 cm^3^ threshold was validated in the SIOP 93-01 and SIOP 2001 datasets as a predictor of outcome within the intermediate-risk histological group at this specific timepoint, with five-year event-free survival of 88% for smaller and 76% for larger tumors [10,11]. Postoperative pathological volume, though measured on the same tumor, captures a different quantity under different conditions; using it for stratification would introduce inconsistency with the evidence base underpinning the SIOP threshold.

The reclassification analysis further illustrates this point: 47% of patients classified as high-risk by radiological volume had postoperative pathology volumes below 500 cm^3^, reflecting the systematic tendency of ex vivo pathological measurement to yield lower values than in vivo imaging. Our data show that using pathology volume would have produced discordant (lower) risk categorization for nearly half of ellipsoid-defined high-risk patients; because actual treatment decisions and outcomes were not evaluated in this study, we cannot state that this would have caused undertreatment or compromised outcomes. This discordance nonetheless underscores that the SIOP 500 cm^3^ threshold is inherently a radiological benchmark, and ex vivo measurements are not just different—they may lead to misinterpretation if used as substitutes.

The moderate-to-strong correlation of intraoperative specimen weight with postchemotherapy radiological ellipsoid volume (r = 0.730, percentage bias +46.6%) is consistent with prior reports in smaller series [14,17]. However, the Bland–Altman analysis reveals a systematic positive bias (weight exceeds radiological volume) with very wide limits of agreement, indicating that weight cannot replace volumetry for quantitative purposes. The biological basis for this discrepancy is well recognized: the nephrectomy specimen includes adherent renal parenchyma, peri-renal fat, and the fibrous pseudocapsule, and necrotic or cystic tumor components. Furthermore, intraoperative weight is influenced by tissue handling, specimen completeness, and the degree of cystic or necrotic change within the tumor. Nevertheless, specimen weight provides a useful immediate intraoperative cross-check against the radiological estimate, and gross discordance may prompt pathological or radiological review.

The uniformity of the ellipsoid–planimetry correlation across stages (r = 0.985–0.998) and histological subtypes (r = 0.996–1.000) is reassuring. We had initially hypothesized that advanced-stage or unfavorable-histology tumors might have more irregular geometry, thereby reducing the accuracy of the ellipsoid approximation; however, our subgroup analyses did not support this assumption.

Pathological specimen weight was excluded from the primary analysis because only 50% of patients had it documented. In the available 30 patients, the correlation with the ellipsoid was high (r = 0.951), but this estimate is unreliable given the degree of missingness and the likelihood that well-documented cases are not representative of the full cohort.

This study has several limitations. The retrospective single-center design introduces the possibility of selection bias and limits generalizability. Because the two radiologists reviewed all studies together and reported a single consensus measurement rather than independent readings, inter- and intra-observer reproducibility for both ellipsoid and planimetric measurements was not formally assessed, and the excellent agreement observed between methods in this study may not generalize across observers or institutions. All radiological measurements were obtained from CT using consistent departmental acquisition protocols throughout the 2020–2024 study period; however, slice thickness, reconstruction interval, and contrast phase were not individually recorded for each examination in this analysis and could not be formally evaluated as a potential source of measurement variability. In addition, because pathological measurements were abstracted from retrospective reports, whether tumor dimensions were recorded before or after formalin fixation was not consistently documented; this uncertainty should be considered when interpreting the negative bias between radiological and pathological volume and the proposed contribution of fixation-related shrinkage. The single patient with a 97% discrepancy between radiological and pathological volume was an influential observation; a sensitivity analysis excluding this case was not performed, and its effect on the reported correlation and limits of agreement should be evaluated in future work. Histological risk group data were not included in this volumetric analysis, and the prognostic interaction between tumor volume and histological subtype—particularly the intermediate-risk group for which the 500 cm^3^ threshold was validated—could not be explored within this cohort. Pathological specimen weight was missing in 50% of the cohort. This missingness reflects an institution-specific documentation limitation that cannot be generalized beyond our center; it limits our ability to characterize the relationship between ex vivo surgical findings and in vivo radiological assessments in this cohort and represents an opportunity for future standardized data collection.

## 5. Conclusions

Postchemotherapy presurgical radiological tumor volume remains the current protocol-specified reference for SIOP risk stratification in WT. Our data show that the ellipsoid formula and radiological planimetry produced highly correlated volume estimates (Pearson r = 0.993) and identical threshold classification in this cohort at the 500 cm^3^ treatment threshold. These findings support continued use of the simpler ellipsoid method in routine practice, pending confirmation in larger, prospectively designed cohorts. Radiological and postoperative (intraoperative specimen weight and pathological) measurements showed systematic and clinically relevant differences; because these methods do not measure the same underlying quantity under the same conditions, and because actual treatment decisions and outcomes were not assessed in this study, postoperative measurements should not be used to revise established radiological SIOP risk classifications outside of prospective validation.

## Figures and Tables

**Table 1:** Patient and disease characteristics (*N* = 60).

Characteristic	*N* or Value
Total patients	60
Median age, years	3.7
Age range, years	0.6–15.2
Female/Male	37/23
Stage I, *n* (%)	16 (26.7)
Stage II, *n* (%)	18 (30.0)
Stage III, *n* (%)	10 (16.7)
Stage IV, *n* (%)	16 (26.7)
FH, *n* (%)	42 (70.0)
BT, *n* (%)	10 (16.7)
DA, *n* (%)	5 (8.3)
FA, *n* (%)	3 (5.0)

Abbreviations, *n*, number; FH, favorable histology, BT, blastemal tumor, DA, diffuse anaplasia; FA, focal anaplasia.

**Table 2:** Descriptive statistics for tumor volume by measurement modality. All radiological measurements obtained postchemotherapy, immediately prior to surgery.

Measurement Modality	*n*	Median (cm^3^)	Range (cm^3^)	>500 cm^3^
Ellipsoid formula (postchemo, presurgical)	60	201	16–1736	15 (25%)
Planimetry (postchemo, presurgical)	60	232	13–1952	15 (25%)
Intraoperative specimen weight (g)	55	344	73–3250	17 (30%)
Pathology volume (postoperative)	54	151	2–682	6 (10%)

**Table 3:** Correlation of each method with the SIOP ellipsoid volume.

Measure	*n*	Pearson r	95% CI	Spearman rho	*p*
Radiology planimetry (cm^3^)	60	0.993	0.988–0.996	0.995	<0.001
Intraoperative kidney weight (g)	55	0.730	0.576–0.834	0.908	<0.001
Pathology tumor volume (cm^3^)	54	0.594	0.388–0.743	0.782	<0.001
Pathological specimen weight (g) *	30	0.951	0.899–0.977	0.915	<0.001

* Exploratory analysis; metric excluded from primary agreement analysis due to 50% missingness in institutional records.

**Table 4:** Ellipsoid vs. planimetry correlation by SIOP stage and histological subtype.

Variable	*N*	Pearson r
Stage I	16	0.985
Stage II	18	0.995
Stage III	10	0.998
Stage IV	16	0.987
BT	10	0.997
DA	5	0.997
FA	3	1.000
FH	42	0.996

Abbreviations: *N*, number; FH, favorable histology, BT, blastemal tumor, DA, diffuse anaplasia, FA, focal anaplasia.

## Data Availability

The data presented in this study are available on request from the corresponding author due to ethical restriction.
